# Effectiveness of a nationwide community pharmacist-led program promoting home blood pressure monitoring on hypertension control and self-management

**DOI:** 10.1038/s41440-025-02422-6

**Published:** 2025-10-23

**Authors:** Yung-Te Chen, Wan-Yu Yeh, Yi-Chun Hu, Shu-Mei Yang, Ren-Hao Pan, Tzu Han Chen, Yu-Fen Liu, Chien-Yuan Wu, Yann-Yuh Jou, Shi-Lun Wei, Chao-Chun Wu, Hao-Min Cheng

**Affiliations:** 1 Department of Internal Medicine, Taipei Veterans General Hospital, Taipei, Taiwan, ROC; 2 Department of Medical Education, Taipei Veterans General Hospital, Taipei, Taiwan, ROC; 3 Health Promotion Administration, Ministry of Health and Welfare, Taipei, Taiwan, ROC; 4 La Vida Tec. Co. Ltd., Taichung City, Taiwan, ROC; 5 Department of Information Management, Tunghai University, Taichung City, Taiwan, ROC; 6 Digital Humanitarian Association, Taichung City, Taiwan, ROC; 7 Health Science and Industry, China Medical University, Taichung City, Taiwan, ROC; 8 Institute of Hospital and Health Care Administration, National Yang Ming Chiao Tung University, Taipei City, Taiwan, ROC; 9 Institute of Public Health and Community Medicine Research Center, National Yang Ming Chiao Tung University College of Medicine, Taipei, Taiwan, ROC; 10 Institute of Epidemiology and Preventive Medicine, College of Public Health, National Taiwan University, Taipei, Taiwan, ROC; 11 PhD Program of Interdisciplinary Medicine (PIM), National Yang Ming Chiao Tung University College of Medicine, Taipei, Taiwan, ROC

**Keywords:** Community, Nationwide, HBPM, Pharmacist

## Abstract

Home blood pressure (BP) monitoring (HBPM) is fundamental to effective hypertension management. Incorporating community pharmacists into care delivery, especially via hybrid case management methods utilizing digital telemonitoring, presents a promising strategy for enhancing BP control and patient self-management. Nonetheless, the efficacy of these interventions at a national level remains inadequately investigated. We performed a nationwide observational study in Taiwan from September to December 2023. A total of 1216 adults with or at increased risk of hypertension were recruited via community pharmacies. Participants were assigned to either digital case management with telemonitoring or traditional pharmacist-led support for 3 months. Outcomes encompassed alterations in BP, alongside hypertension-related knowledge, attitudes, and self-management behaviors. In hypertensive participants, systolic and diastolic BP decreased from 133.2 ± 12.3 mmHg to 129.8 ± 19.3 mmHg (*P* = 0.002) 83.1 ± 19.2 mmHg to 79.7 ± 9.0 mmHg (*P* < 0.001) over a period of 3 months, respectively. The questionnaire achieved a response rate of 94%, with notable enhancements observed in knowledge (+1.2 points), attitude (+4.2 points), and behavior (+3.5 points) scores (all *P* < 0.001). Older adults, individuals with diminished educational qualifications, and residents of less urbanized regions exhibited more significant improvements. No notable differences were detected between digital and non-digital case management in BP or behavioral outcomes. A nationwide hybrid intervention promoting HBPM via community pharmacist-led case management resulted in substantial enhancements in BP control and self-management outcomes. Moreover, the method was especially advantageous for socially disadvantaged groups. These findings endorse incorporating pharmacist-led hybrid care models into national hypertension management strategies.

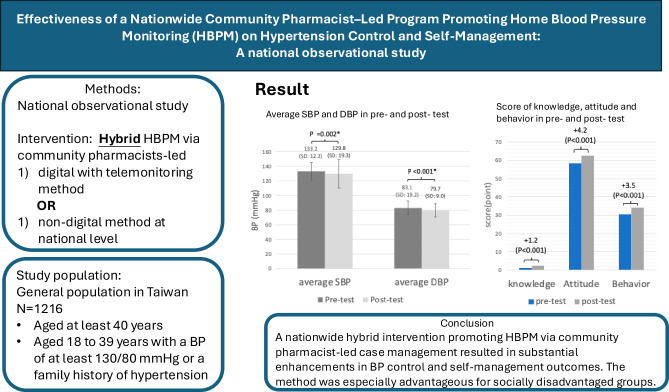

## Introduction

Hypertension is the top risk factor for cardiovascular disease and stroke in Taiwan, while heart, cerebrovascular, and hypertensive diseases were the 2nd, 4th, and 6th leading causes of death in Taiwan in 2024, respectively; since 2010, all three have been listed in Taiwan’s top ten causes of mortality. Managing hypertension is crucial to lowering these risks and preventing related complications.

Financed by the Health Promotion Administration, Ministry of Health and Welfare in Taiwan, our team executed the National Health and Nutrition Survey in Taiwan from 2013 to 2016, which indicated an overall hypertension awareness rate of 72.06%. The treatment rate for individuals aware of hypertension was 89.35%. The overall control rate for hypertensive individuals was 49.90% [1]. Despite awareness, treatment, and control rates surpassing global averages from 1990 to 2019 [2], substantial opportunities for improvement remain.

According to the 2022 Guidelines of the Taiwan Society of Cardiology and the Taiwan Hypertension Society for the Management of Hypertension [3], home BP monitoring (HBPM) based on the 7-2-2 protocol is recommended as the standard method for diagnosis and long-term management due to better prognostic value and clinical utility [4]. Consequently, hypertension telemonitoring management through HBPM shows promise in improving BP among the general population. Moreover, along with the Dietary Approaches to Stop Hypertension (DASH), numerous hypertension guidelines advocate lifestyle modifications such as sodium restriction, moderation of alcohol intake, weight reduction, cessation of smoking, dietary adjustments, and the incorporation of exercise (SABCDE) for effective BP management, and can be incorporated into hypertension telemonitoring programs [5, 6].

Ideal remote hypertension management requires HBPM, data integration by technology, and team management with pharmacists and physicians. Previous meta-analyses on the effect of hospital or community pharmacist intervention, including medication education reveals increased systolic BP (SBP) and diastolic BP (DBP) reduction, medical knowledge, awareness, and adherence to medical intervention [7–9]. Similarly, evidence has shown that digital case management by hospital-based health workers can effectively control BP [10].

The Taiwan National Health Service’s 2020 Guidelines for Cardiovascular Disease Prevention and Care classify community pharmacist-guided interventions as Class IB recommendations for BP control. Additionally, BP telemonitoring, indicating BP readings are automatically summarized and sent to the care provider, is proved to enhance BP control [11–14]. Moreover, telemonitoring combined with pharmacist-led interventions, including medication adjustments based on standardized protocols, has demonstrated significant BP reductions and improved adherence to medical support and health behaviors in previous randomized controlled trials [10, 15]. Furthermore, Lau’s findings support the efficacy of telemonitoring combined with community pharmacy case management in BP control [16].

Nevertheless, previous studies have predominantly focused on patients with established or uncontrolled hypertension, thereby resulting in significant gaps in comprehending how telemonitoring and pharmacist-led digital interventions could advantage normotensive individuals at elevated risk for future hypertension. Moreover, investigation into the impact of these interventions on participants’ hypertension-related knowledge, attitudes, and behaviors (KAB) is insufficient, especially regarding adherence to and understanding of the 7-2-2 protocol for HBPM, particularly on a national scale. Whether interventions utilizing digital tools substantially improve knowledge of recommended measurement times, willingness to comply with self-monitoring schedules, and proactive implementation of lifestyle changes as specified by the SABCDE and DASH guidelines is uncertain. Addressing these deficiencies is crucial to extend preventive strategies beyond individuals already diagnosed with hypertension, ultimately fostering early intervention and efficient risk management across wider populations.

Additionally, non-digital interventions remain indispensable, as not all individuals can rapidly adapt to digital tools. Consequently, relying solely on digital interventions and telemonitoring may yield suboptimal outcomes in the general population. Accordingly, a hybrid model that combines digital interventions with non-digital case management is more suitable for national implementation.

To address these issues, we conducted, to the best of our knowledge, the first nationwide campaign in Taiwan to assess the effects of community pharmacy-based interventions that combine digital case management with telemonitoring and non-digital case management on HBPM within the general populace. The efficacy of the intervention was evaluated by examining alterations in BP, hypertension-related KAB, along with compliance and comprehension of the 7-2-2 home monitoring protocol.

Point of view

- Clinical relevance
  National implementation of a hybrid model of digital case management with telemonitoring and non-digital approaches by community pharmacies potentially improves blood pressure control and enhance knowledge, attitudes and behaviors related to hypertension self-management among adults with hypertension and high-risk adults, especially for individuals of older age or lower socioeconomic status.
- Future direction
  Further randomized control studies primarily exploring the comparative benefits for knowledge, attitude, and health behavior change between digital with telemonitoring and non-digital case management by community pharmacies among individuals who are normotensive but with risk of developing hypertension is warranted.
- Consideration for the Asian population
  The finding of the present study offers a potential national modality to enhance hypertension control for those Asian countries with high hypertension prevalence or suboptimal hypertension control rates.

## Methods

### Study design

This study is a nationwide implementation and observational research conducted in Taiwan from September to December 2023. Based on their preference, all participants received telemonitoring with either digital or non-digital case management from a pharmacist. Questionnaires were distributed to participants before and after case management.

### Participant recruitment

Participants were recruited through an invitation from pharmacists. A total of 1216 participants who were (1) aged at least 40 years or (2) aged 18 to 39 years with a BP of at least 130/80 mmHg or a family history of hypertension were included. Those who had low willingness to participate, were not able to perform regular BP monitoring at home or at a pharmacist, or visit a pharmacy regularly were excluded.

### Pharmacist participation

This study involved 54 community pharmacies nationwide, which were mandated to supply onsite BP monitors, offer both application and paper recording options, and be prepared to deliver case management and consultations. The HBP telemonitoring application was launched on the WaCare platform (https://www.wapro.live), which was developed by La Vida Tec Co., Ltd in Taiwan. WaCare connects healthcare professionals and a network of caring family and friends. By leveraging an AI engine, machine learning algorithms, and natural language processing, the platform integrates personal medical records, government open data, and wearable device information to model individual behaviors and predict health risks. Proactive risk alerts are used to prompt online consultations and caring interventions, thus enabling early mitigation of potential health issues.

All the participating pharmacist received training program, which includes the framework, process and meaning of HBMP and module of BP management, health education, the role of community pharmacist and introduction of WaCare platform. Pharmacists completed a pre-test prior to the training program and a post-test afterward to evaluate their performance. The correct response rate for each post-test question exceeded 70%, indicating a high level of pharmacist competency following the training.

### Questionnaire design and data collection

The data were divided into two components: questionnaire and BP data.

The questionnaire was designed based on the 2011 study on the effectiveness of a telehealth care system for self-management behaviors in hypertension patients and the 2022 Home-Based BP Case Management Service Model Development Program proposed by the Health Promotion Administration, Ministry of Health and Welfare, Taiwan [17].

The pre- and post-test questionnaire consisted of three categories of questions, covering knowledge of hypertension, 7-2-2 principles for HBPM, attitude toward self-management of hypertension, and health behaviors. (Each category contains three, seven, and eight questions, respectively). In the knowledge category, responses evaluated by the pharmacist as “completely correct” are considered correct, while “partially correct” or “unknown” responses are considered incorrect. In the attitude category, each question is scored from 1 (completely disagree) to 10 points (completely agree). In the health behavior category, the scoring method for each question is as follows: how frequently participants have engaged in the following health behaviors each week is rated on a scale of 1–5 for the past three months (1 = did not do it, 2 = did it 1–2 days, 3 = did it 3–4 days, 4 = did it 5–6 days, and 5 = did it all 7 days).

The internal consistency reliability of the questionnaire was evaluated via Cronbach’s alpha based on pre-test results, with KAB. Cronbach’s alpha of the three scale questions was all over 0.8. The details of the questionnaire are presented in Supplementary Table 1.

BP data were collected at two different time points. The initial screening BP measurement was conducted at the pharmacy during participant recruitment. BP measurements were obtained via HBPM at baseline and three months post-intervention. The measurement taken at the pharmacy was used to classify participants’ baseline BP status within the demographic characteristics. By contrast, BP data obtained via HBPM were used to assess changes in BP following the intervention. BP data, including SBP, DBP, and heart rate, were collected at least four times a day, twice in both the morning and at night. As some people may not have a BP monitor at home, participating pharmacies provided BP monitors for people to measure their BP during business hours to complete BP records.

### Telemonitoring and case management by pharmacists

At the beginning of the study, all participants visited a community pharmacy, during which they completed a pre-test questionnaire to collect their demographic data, educational level, hypertension history, and evaluate their knowledge, attitudes, and health behaviors. Subsequently, the pharmacist provided health education about the 7-2-2 protocol for HBP monitoring, SABCDE lifestyle modifications, and DASH diet.

The participants were then stratified into digital or non-digital case management groups based on their preference. Some received 3-month HBPM with digital case management, and the others received 3-month HBPM with non-digital case management based on their preference. In the digital case management group, BP was recorded in an app. Monitoring and management were administered through the app, which offered reminder, real-time feedback, and consultation to the pharmacists. Upon entry of their BP into the app, the data were automatically relayed to the pharmacies. The platform subsequently analyzed the BP data and notified participants who do not adhere to the 7-2-2 protocol or exhibit abnormal BP readings. Additionally, case managers could track patients’ BP via the smartphone app. The aforementioned process is illustrated in Supplementary Fig. 1.

A paper-based BP record was conducted in the non-digital case management group. Patients were required to document their BP and present it at the pharmacy during each visit. Monitoring and management were conducted via telephone and SMS reminders, along with feedback during follow-up. Patients were required to make monthly visits to the pharmacist.

Participants who had abnormal BP during telemonitoring will receive health education or be referred to specialists for further management based on the principles in Supplementary Table 2. Referral is administered through the app in the digital group and via phone or during pharmacist visit in the non-digital group.

**Fig. 1:**
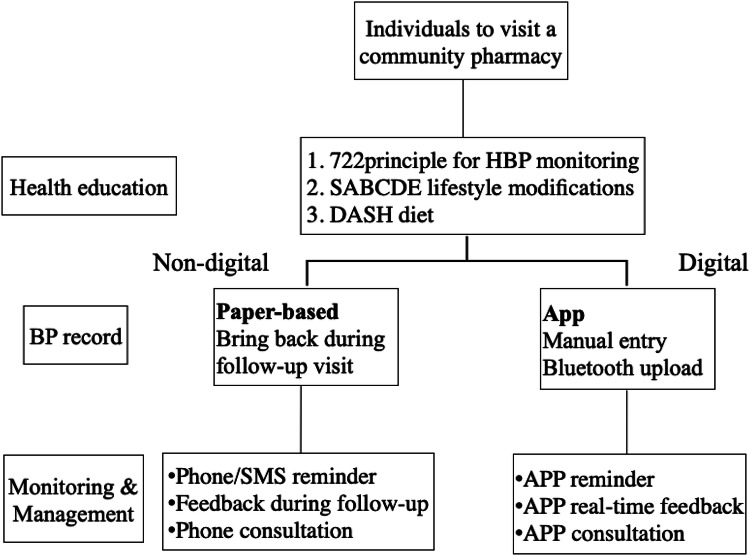
Flow chart of the study process

The study procedure is illustrated in Fig. 1.

The outcomes were assessed following a three-month intervention. Baseline pharmacy BP data and three months of home BP measurements were obtained during the three-month follow-up period. The participants were given post-test questionnaires to evaluate the impact of the telemonitoring with digital or non-digital case management on their KAB related to hypertension, 7-2-2 protocol, and self-management of hypertension. Difference in BP and KAB were carefully analyzed. Achieving the 7-2-2 protocol was defined as a participant measuring their BP at least three times on three different days within one week, at least once during the entire study period [3].

### Compliance with 7-2-2 protocol and BP difference between pre- and post-test

Compliance with the 7-2-2 protocol was defined as documenting BP measurements on three separate days within a one-week period, with each day consisting of two readings each in the morning and evening [3]. Participants were considered compliant if this protocol was followed at least once during the entire study period. The BP difference between pre- and post-test was defined as the difference between the average BP on the last day with BP recording and that on the first day with BP recording.

The classification of urbanization and digitalization of pharmacy location were elucidated in Supplementary material.

### Statistical analysis

All analyses were performed using SPSS (IBM, US, version 29&30), R (Version 4.4.1), and Prism 10. Continuous data are presented as mean (SD), while categorial data are shown as N (%).

For sample size calculation, a mean SBP reduction of 1.5 mmHg was assumed as the effect size. The standard deviation of the pre–post difference was set at 12 mmHg, based on prior East Asian trial of blood pressure changes following intervention in 3 months [18].Under a two-sided significance level of 0.05 and a power of 0.80, the required sample size was calculated to be 502 participants. Considering a potential attrition rate of 15–20%, the adjusted target sample size was set at approximately 590–628 participants to ensure adequate power.

Owing to the existence of BP outliers in BP data collected at the pharmacy during participant recruitment, BP values exceeding three standard deviations from the mean were excluded from the BP analysis regarding classifying participants’ baseline BP status within the demographic characteristics. Missing data on attitudes or behaviors were filled with the average score since participants have a tendency to display specific attitude and behavior. Missing data on knowledge remained blank during analysis. The proportion of imputed data relative to the total dataset in each question is ≤2% (Supplementary Tables 6, 7).

Participants were excluded from BP analysis if either pre- or post-test BP data were completely missing. Likewise, data were excluded from analyses of knowledge, attitude, and health behaviors if either the pre- or post-test questionnaire data were completely missing. We also compared complete-case results with mean-imputed values, which showed negligible differences, thus indicating robustness of findings.

The paired-t test was used to analyze changes in BP, attitudes, and health behaviors before and after the intervention. Difference in score or BP was defined as values of post-test score or BP minus pre-test score or BP, respectively. The McNemar test was used to evaluate the changes in knowledge before and after the intervention. χ² test was used to evaluate the association between choice of digital or non-digital case management with compliance with the 7-2-2 protocol.

A linear mixed-effects model with repeated measures of BP as the dependent variable was constructed to examine whether lifestyle or drug adherence change during the study contributed to BP control in this study. Independent variables included the frequency of exercise, reduced salt intake, vegetable consumption, reduced alcohol intake, medication adherence, hypertension status, age, and sex. Interaction terms between time and each independent variable were incorporated to assess differential effects over time.

After excluding participants with completely lacking either pre- or post- test questionnaire responses or home BP data, determinants affecting the preference for digital versus non-digital case management were examined using multiple logistic regression analysis. An odds ratio (OR) exceeding 1 signifies an increased probability of selecting non-digital case management compared to digital case management.

Subgroup analysis was conducted to examine differences in changes in BP and KAB across groups with distinct demographic characteristics. Differences in post-test scores of KAB within each subgroup and magnitude of pre-to-post-intervention score changes within certain subgroups were analyzed via analysis of variance. The subgroup analysis of SBP and DBP change post-intervention was adjusted for baseline BP status using analysis of covariance (ANCOVA). *P*-values for the differences in BP change within subgroups were also computed.

The effect size of digitalized case management was defined as the difference between pre- and post-test SBP, DBP and scores in KAB for the digitalized case management group, minus the corresponding difference in SBP, DBP and scores in KAB for the non-digitalized group. ANCOVA was also performed to calculate the interaction between the effect sizes of digital and non-digital case management on KAB change across subgroups as sex, age, educational level of participants, urbanization, and digitalization of pharmacy location.

A post hoc analysis was performed to calculate the ratios of participants receiving non-digital to digital case management in different urbanized regions. A significant difference was defined as a *p*-value of less than 0.05.

## Results

### Participant characteristics and questionnaire reliability

A total of 1216 participants were recruited for this study, achieving a response rate of 94% to the questionnaire. After excluding those with completely missing data on BP or questionnaire data, a total of 797 and 829 participants who didn’t completely miss pre-test or post-test BP data, and who didn’t completely lack pre- or post-test questionnaire data were enrolled, respectively. The clinical characteristics of the 1216 participants are summarized in Table 1. Males constituted 36.5% of the study population, with an average age of 56.2 years. Approximately 43% of the participants possessed university, college, or higher academic degrees. The baseline BP recorded by the pharmacist was 122.3/76.4 mmHg, with the hypertensive demographic constituting 47.9% of the total population.

**Table 1:** Overall clinical characteristics of participants

Variables	*N* = 1216
Gender	
Male	444 (36.5%)
Female	611 (50.3%)
Missing	161 (13.2%)
Age group	
<40 y/o	168(13.8%)
40 to 64 y/o	518(42.6%)
≥65 y/o	374(30.8%)
Missing	156(12.8%)
Education	
Elementary school or below	142(11.7%)
Junior high school	101(8.3%)
High school/Vocational high school	277 (22.8%)
University/College	455 (37.4%)
Master	74 (6.1%)
PhD	5 (0.4%)
Missing	162 (13.3%)
Physical examination	
Body Mass Index(kg/m^2^)	24.337 (4.1)
Waist circumference(cm)	83.6 (13.5)
SBP at baseline(mmHg)	122.3 (15.6)
DBP at baseline(mmHg)	76.4 (11.0)
Hypertension *N* (%)	493(40.5%)
Family history of hypertension	713 (58.6%)
BP monitoring before	
Have a BP monitor at home, *n* (%)	773 (75.7%)
Self-monitoring BP, *n* (%)	527 (51.6%)
Through app or computer	145(14.2%)
Through handwriting	382(7.4%)

Categorial data are presented as *N* (Percentage in %); continuous data, such as Body Mass Index (BMI), waist circumference, SBP at baseline, and DBP at baseline, are presented as mean (standard deviation). Hypertension is defined as BP ≧ 130/80 mmHg

### Selection between digital and non-digital case management

A logistic regression analysis of factors influencing the choice between digital and non-digital case management, excluding participants with incomplete data, is presented in Table S3. Age, educational level, and urbanization level of participating pharmacy location were significantly correlated with the selection of management type. Older adults exhibited a higher propensity for selecting non-digital case management, whereas elevated educational attainment and urbanization of participating pharmacy location correlated with an increased likelihood of opting for digital management. Conversely, sex, digitalization level of participating pharmacy location, and pre-intervention hypertension status exhibited no significant correlation with the chosen type of case management. These findings indicate that demographic and contextual factors, especially age, education, and urbanization level of participating pharmacy location, affect preferences for digital health services. No correlation was observed between the choice of digital or non-digital case management and compliance with the 7-2-2 protocol (*P* = 0.976).

### Outcome following intervention of combined telemonitoring and community pharmacy case management

**Fig. 2:**
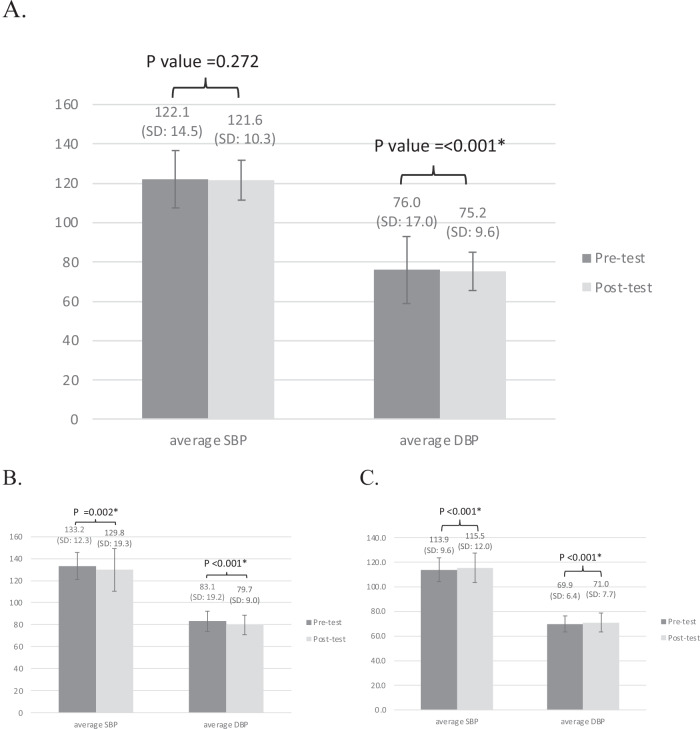
Average systolic and diastolic blood pressure (SBP, DBP, respectively) with standard deviation (SD) before and after intervention. **A** indicates analysis for general population (*N* = 797). **B**, **C** indicates analysis for the hypertension group (*N* = 355) and normotension with high-risk group (*N* = 422), respectively. The unit of BP is mmHg. The gray vertical lines indicate the 95% confidence interval (CI)

The change in BP before and after the intervention is illustrated in Fig. 2. An insignificant reduction in SBP (122.1 ± 14.5 mmHg to 121.6 ± 17.0 mmHg, *p-*value = 0.272) is observed, whereas DBP decreased significantly after intervention (76.0 ± 10.3 mmHg to 75.2 ± 9.6 mmHg, *p-*value <0.001). When the general population was stratified into hypertension and normotension with high-risk groups, the intervention significantly reduced SBP (133.2 ± 12.3 mmHg to 129.8 ± 19.3 mmHg, *p-*value = 0.002) and DBP (83.1 ± 9.2 mmHg to 79.7 ± 9.0 mmHg, *P* < 0.001) in the hypertension group, while no decrease of SBP (113.9 ± 9.6 to 115.5 ± 12.0, *P* < 0.001) or DBP (69.9 ± 6.4 to 71.0 ± 7.7, *P* < 0.001) in normotension was observed in the high-risk group.

The analysis for repeated blood pressure in relation to lifestyle and drug adherence change through linear mixed model revealed baseline hypertension status, male sex and age were significantly associated with overall higher blood pressure levels (main effect, *p* < 0.001) whereas other lifestyle factors, including exercise, diet, and alcohol abstinence, were not. The interaction analysis revealed that only the interaction between medication adherence and time was significantly correlated with blood pressure reduction (time × adherence, *β* = −8.397, p < 0.001). No interaction was found between lifestyle factors and time (*p* = 0.3–0.6). (Table S3)

**Fig. 3:**
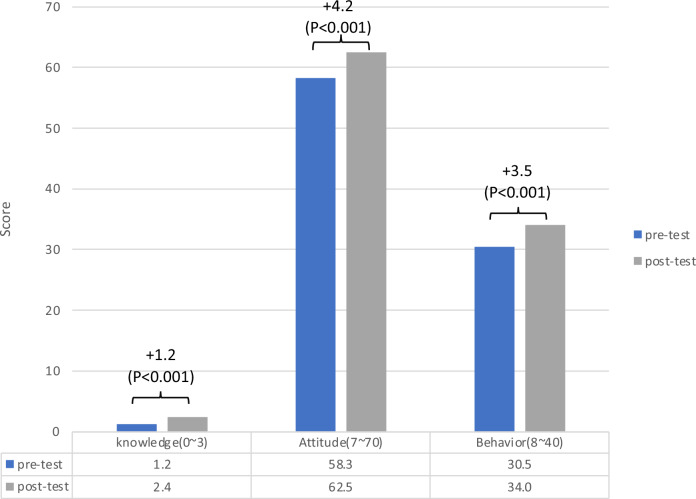
Score of knowledge, attitude, and behavior in pre-and post-test. (N = 829) Difference in scores between pre and post-test in each category is displayed above the bar in each category

Following the intervention, a significant improvement in KAB related to self-management for hypertension was observed. Knowledge, attitude, and behavior scores increased by 1.2, 4.2, and 3.5 points, respectively (all *P* < 0.001, Fig. 3). The corresponding standard mean difference of knowledge, attitude, and behavior are 1.04, 0.04, and 0.09, respectively.

Specifically, knowledge of the 7-2-2 principle, willingness to follow the 7-2-2 principle, and the likelihood of seeking professional assistance for BP control and improvement of nearly all health behaviors significantly increased (Supplementary Tables 5-7).

### Subgroup analysis

No significant change of SBP following intervention after stratification by sex, age, educational level, urbanization level, and digitalization level of participating pharmacy location and adjustment of baseline BP status was observed, while DBP decreased in the stratified population of male, female, aged ≥40 years old, with medium educational level, and pharmacies in regions with medium urbanization and digitalization (see Fig. S2). Nevertheless, no significant differences were observed within subgroups across these categories.

**Fig. 4:**
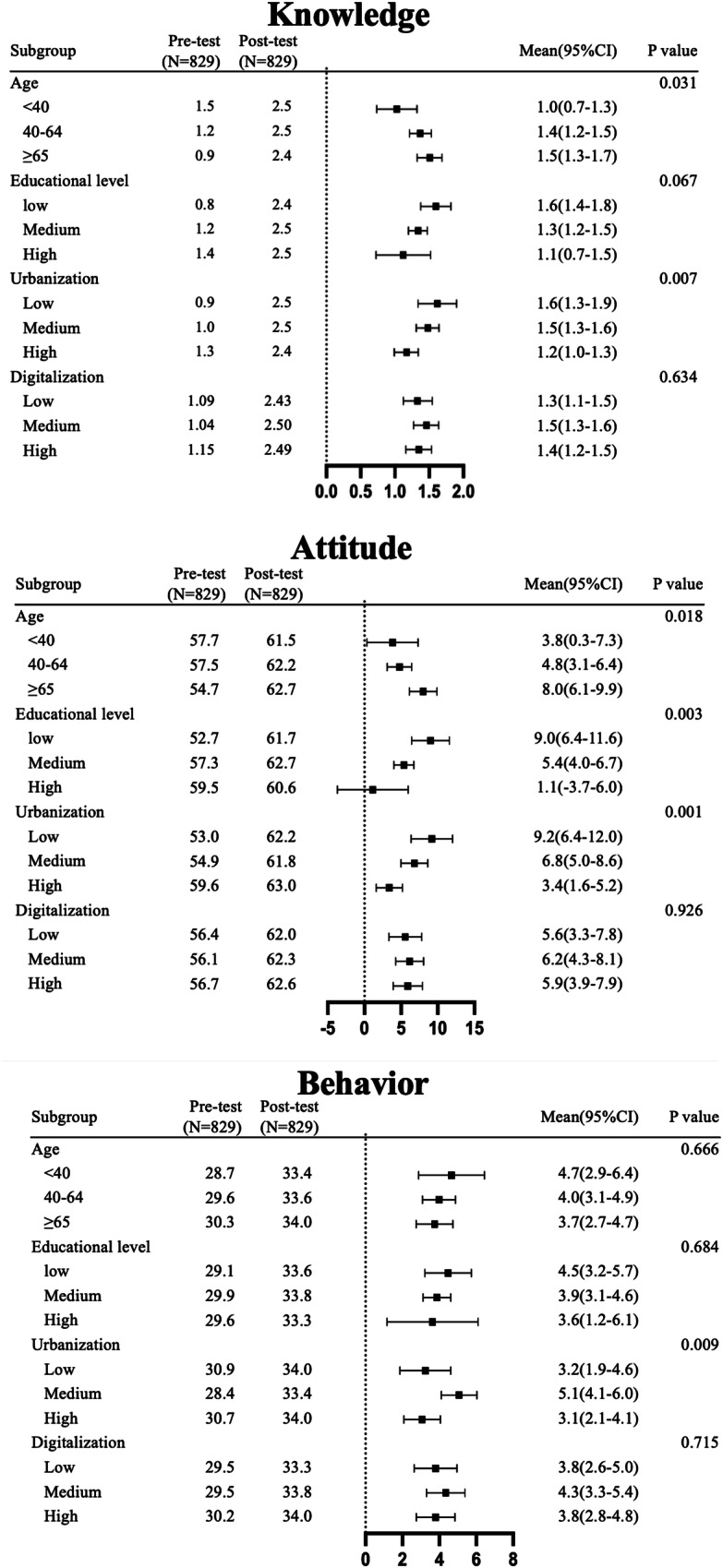
Subgroup analysis of knowledge, attitude and behavior score before and after intervention (Missing data were imputed with mean value). “Pre-test” and “Post-test” refer to scores within each subgroup before and after the intervention, respectively. ‘Mean’ denotes the average difference between post-test and pre-test scores, presented with the corresponding 95% confidence interval (95% CI). Age is measured in years. The black dots represent the average difference between post-test and pre-test scores for each subgroup, while the horizontal lines indicate the 95% CI. The scale beneath the forest plot indicates the score values of knowledge, attitude or behavior

However, except for attitude scores in participants with high educational attainment, significant increases in knowledge, attitude, and behavior scores were observed across most subgroups. No significant differences were found among post-test scores of KAB within each subgroup. Nevertheless, the magnitude of pre-to-post-intervention score changes differed significantly within certain subgroups, (Supplementary Tables 8, 9, 10; Fig. 4). For knowledge score, a significant difference was found in age and urbanization, with people ≥65 years old and living in lower urbanized regions exhibiting the greatest score improvement. For attitude score, a significant difference was observed in age, education and urbanization subgroup, with people ≥65 years old, having lower educational attainment and living in lower urbanized regions displaying the greatest score improvement. For behavior score, a significant difference was observed in the urbanization subgroup, with the greatest score improvement among people living in regions with medium levels of urbanization.

### Effect size and interaction between digital and non-digital case management

The effect size of digital vs. non-digital case management on SBP, DBP, knowledge, attitude, and behavior scores are depicted in Supplementary Table 11. In addition, the interaction between case management type and baseline participant characteristics is presented in Supplementary Fig. 3. No significant advantage of digital case management over non-digital was observed for SBP, knowledge, and attitudes improvement. However, digital case management was associated with a slightly greater reduction in DBP compared to non-digital case management (mean difference: −1.1 mmHg, *P* = 0.045). Conversely, non-digital case management yielded a mildly greater behavior improvement than digital case management (mean difference: −1.7, *P* = 0.047). A significant interaction was found between case management preference (digital vs. non-digital) and sex for knowledge scores (*P* = 0.043). Additionally, a significant interaction was observed between case management preference and urbanization level for behavior scores (*P* = 0.002). Notably, females, individuals aged 40–64 years, participants with medium educational levels, and those residing in highly urbanized areas displayed greater improvements in behavior scores when using non-digital case management.

As presented in the supplementary material (Tables S6–S10), the sensitivity analysis reveals that imputing mean values for missing attitude and health behavior scores did not significantly affect the results.

## Discussion

This study represents the inaugural extensive national campaign and implementation analysis of the effects of telemonitoring, which was administered through both digital with telemonitoring and non-digital pharmacist-led case management, on BP control and hypertension-related KAB in hypertensive and normotensive individuals at heightened risk of developing hypertension. After a 3-month intervention conducted by community pharmacists, participants demonstrated significant improvements. Notably, significant decreases were observed in both SBP and DBP in individuals with hypertension, accompanied by significant improvements in KAB scores pertaining to hypertension self-management, including recognition of the 7-2-2 protocol. The intervention was especially advantageous for older individuals with lower educational attainment living in less urbanized areas, while digital and non-digital case management exhibited comparable effects on BP control and self-management. Moreover, these findings highlight the effectiveness of community pharmacy-based case management combining digital and telemonitoring technologies and non-digital approach as a proactive, preventive strategy for early detection and risk mitigation in at-risk populations.

Since the majority of participants were normotensive at baseline, no significant reduction in SBP was observed. However, the intervention successfully maintained BP levels in individuals at risk of developing hypertension. Additionally, a significant decline in DBP was observed among older participants. By contrast, significant improvements were noted in KAB following the implementation of digital case management with telemonitoring by community pharmacists. Specifically, participants’ recognition of the 7-2-2 protocol increased substantially, with the correct response rate rising from 30.1% before the intervention to 79.5% thereafter. Among the various attitude-related improvements, participants’ willingness to share BP information with others displayed the most notable change. Moreover, knowledge of the DASH diet also improved. These promising effects can be attributed to the telemonitoring system, which integrates a mobile application with automated feedback, including encouragement, recommendations, and notifications. In the linear mixed model analysis assessing the impact of lifestyle factors and drug adherence on BP change, only drug adherence positively influenced the trajectory of BP over time, indicating that drug adherence, rather than lifestyle modification, is the principal factor contributing to BP reduction in this study. Regarding knowledge acquisition, the increase in knowledge scores among older participants was significantly greater than that observed in younger individuals (*P* = 0.031). Similarly, participants residing in less urbanized areas experienced a greater increase in knowledge scores compared to those in highly urbanized areas (*P* = 0.007). Since post-test knowledge scores did not significantly differ within age or urbanization subgroups, community pharmacist-led hybrid case management combining a digital approach with telemonitoring and non-digital approach may effectively enhance hypertension self-management knowledge among older adults and individuals living in lower urbanized areas to a similar extent as their younger and more urbanized counterparts. Regarding attitude, older individuals, participants with lower educational levels, and those residing in less urbanized areas exhibited improvements to the degree comparable to those who were younger, had higher educational attainment, and lived in highly urbanized regions. Likewise, participants from moderately urbanized regions demonstrated behavioral improvements to a similar extent to those from both low- and high-urbanization areas. These findings underscore the importance of combined digital and non-digital case management by community pharmacists. The intervention enables individuals with lower socioeconomic status to achieve comparable improvement of knowledge and attitude as those with higher socioeconomic status, ultimately reinforcing its potential as an equitable and effective strategy for hypertension self-management.

The field of digital case management has expanded considerably in recent years. For example, Mobile Health (mHealth) is the support and delivery of health services through mobile devices (mobile phones, patient monitoring devices, personal digital assistants, and other wireless devices). The technology behind it includes but is not limited to short messaging systems (SMS), global positioning systems, voice calls, Bluetooth, and mobile phone applications. These systems can not only collect patient’s BP data automatically but also send automatic feedback of recommendation, encouragement, and reminders according to patient’s BP value [19]. Several previous studies explore the BP-lowering effect of telemonitoring with pharmacist intervention. In those studies, pharmacist intervention requires face-to-face visit, phone, app, web, or a combination of these media [10, 15, 20, 21]. Previous studies and meta-analyses have demonstrated the beneficial effect on BP control, increasing medication adherence, knowledge, self-care behavioral outcomes and psychosocial well-being by mobile health or electronic health interventions compared to standard care [22–24]. The app in our research encompasses multifactorial functions as mentioned above. Moreover, preferences for digital case management with telemonitoring were observed among individuals of higher educational levels, residing in more urbanized areas, and of younger age. Thus, such management should theoretically achieve better BP control and KAB improvement toward self-management for hypertension. However, opting for digital case management did not necessarily translate to substantial SBP reduction or enhanced KAB related to hypertension self-management compared to non-digital case management (Supplementary Table 11, Supplementary Fig. 3), which is in contrast to previous research [22–24].

Importantly, nearly all previous studies were randomized controlled trials, whereas ours was a national observational study in which participants received either digital or non-digital case management based solely on their personal preference. Consequently, sociodemographic factors needed to be considered in the analysis. Accordingly, a plausible explanation for our findings is that individuals who opted for non-digital case management, often due to unfamiliarity with digital technology, were generally older, had lower educational attainment, and resided in less urbanized areas. Despite these characteristics, they may have exhibited stronger intrinsic motivation for behavioral and knowledge-related changes, thereby contributing to improved self-management of BP and achieving acceptable BP control. Nevertheless, individuals residing in highly urbanized regions demonstrated greater behavioral improvements when receiving non-digital case management. This may be partially explained by a coincidentally higher level of pharmacist proactiveness in promoting hypertension self-management in these areas. Furthermore, post hoc analysis revealed that the ratios of participants receiving non-digital to digital case management were 2:1, 3:1, and 7:1 in highly, moderately, and less urbanized regions, respectively. This distribution may partly explain the greatest improvement in behavior scores observed in moderately urbanized regions.

From a national policy standpoint, while the promotion of digital case management is warranted due to its myriad benefits, such as enhancing hypertension self-management, mitigating infection risk during pandemics by reducing in-person interactions, and lowering travel expenses for individuals in remote areas, simultaneous implementation of both digital and non-digital case management strategies may be crucial for optimizing self-BP management across varied populations.

### Limitations

This study has several limitations. First, as an observational study, our findings may be subject to confounding due to the lack of randomization. The imbalance in group sizes was also observed, with the majority of participants, especially those from less urbanized areas, opting for non-digital case management. This self-selection may have introduced selection bias and limits the ability to draw causal inferences, although adjustments were made for baseline covariates. Second, the 7-2-2 protocol employed in this study, in which BP measurements are taken over three separate days within a one-week period, with two readings in the morning and two in the evening each day, differs from the standard definition, which involves consecutive BP measurements for seven days, taken twice daily (morning and evening) with two readings each time. Therefore, the extent to which our findings on self-management regarding 7-2-2 protocol can be extrapolated to the standard real-world protocol remains uncertain. Third, questionnaire responses may have been influenced by social desirability bias, as they may have provided answers they believed to be more socially acceptable. Fourth, the proactiveness and professionalism of the pharmacists may be substantially heterogeneous, as they were recruited rather than randomly assigned, potentially influencing the effectiveness of education delivery and subsequent blood pressure monitoring. Nevertheless, the high correct response rate on the post-training test suggests that their level of professional competence was at least adequately ensured.

Despite these limitations, our study still presents strong evidence supporting the effectiveness of the hybrid model of digital case management with telemonitoring along with non-digital case management in BP control and enhancing high-risk adults’ KAB related to self-management of hypertension, including the 7-2-2 principle. Our study also represents the largest trial involving nationwide community pharmacy participation combined with digital and non-digital case management. More importantly, as a national campaign and implementation study, our research includes individuals with comorbidities who might typically be excluded from randomized controlled trials or cohort studies, thus making our findings more reflective of real-world conditions.

Further randomized control studies could explore the comparative benefits for knowledge, attitude, and health behavior change especially for complete 7-2-2 protocol adherence between digital with telemonitoring and non-digital case management by community pharmacies among individuals who are normotensive but with risk of developing hypertension.

### Asian perspectives

Hypertension control rate is highly heterogeneous in Asian with poor control rate particularly at developing Asian countries, attributed to low awareness for hypertension and poor medication adherence at individual level. At national level, contributing factors include inadequate implementation of hypertension management policies, insufficient follow-up strategies, and unequal distribution of healthcare resources in rural regions. Nevertheless, telemedicine and remote monitoring technologies have made healthcare more accessible to people living in remote areas who would otherwise have limited access to healthcare services [25]. In this context, our present study demonstrates that community pharmacist-led case management is valid in hypertension management on a national level and supports its integration into routine care practices, offering a feasible modality for government-led hypertension control routine care practices in Asia.

## Conclusion

The national implementation of a hybrid model of digital case management with telemonitoring and non-digital approaches by community pharmacies has the potential to improve BP control and enhance KAB related to hypertension self-management among adults with hypertension and high-risk adults, with particular benefits for individuals of older age or lower socioeconomic status. In combination with digital and non-digital approaches, community pharmacist-led case management proves effective in hypertension management on a national level and supports its integration into routine care practices.

## Supplementary information

Supplementary information
