# Supplementary material for: Effectiveness of a nationwide community pharmacist-led program promoting home blood pressure monitoring on hypertension control and self-management
Source: Hypertens Res. 2025 Oct 23;49(2):315–27. doi: 10.1038/s41440-025-02422-6 (PMC12823383; doi:10.1038/s41440-025-02422-6)
Supplement: Supplementary file 1 — Supplementary information [file 41440_2025_2422_MOESM1_ESM.docx]

**Supplemental material**

**Effectiveness of a Nationwide Community Pharmacist–Led Program Promoting Home Blood Pressure Monitoring on Hypertension Control and Self-Management**

Yung-Te Chen, MD,^1^ Wan‑Yu Yeh, Ph.D.,^2*^ Yi-Chun Hu,^3^ Shu-Mei Yang, Ph.D.,^2^ Ren-Hao Pan, Ph.D.,^4,5,6^ Tzu Han Chen,^7^ Yu-Fen Liu,^3^ Chien-Yuan Wu,^3^ Yann-Yuh Jou,^3^ Shi-Lun Wei,^3^ Chao-Chun Wu,^3^ Hao-Min Cheng, M.D., Ph.D.^2,8,9,10,11^

CORRESPONDENCE:

Hao-Min Cheng, M.D., Ph.D.

Email: [hmcheng@vghtpe.gov.tw](mailto:hmcheng@vghtpe.gov.tw)

**Content**

[I. Supplemental methods 4](#_Toc209310033)

[Supplementary Table 1. Questions in questionnaire 4](#_Toc209310034)

[Supplementary Figure 1. Schematic diagram of the HBP telemonitoring application launched on the WaCare platform 5](#_Toc209310035)

[Supplementary Table 2. Criteria for the pharmacy to refer patients to the clinics 7](#_Toc209310036)

[Questionnaire design 8](#_Toc209310037)

[Classification of urbanization and digitalization of pharmacy location 8](#_Toc209310038)

[II. Supplemental Results 9](#_Toc209310039)

[Supplementary Table 3. Linear mixed-effects model of systolic blood pressure over time and its association with lifestyle changes and medication adherence 9](#_Toc209310040)

[Supplementary Table 4. Multiple Logistic Regression Analysis for Factors Associated with digital or non-digital case management 10](#_Toc209310041)

[Supplementary Table 5. Proportion of correct responses for knowledge before and after intervention 10](#_Toc209310042)

[Supplementary Table 6. Attitude change before and after intervention (Missing value was imputed with mean value.) 12](#_Toc209310043)

[Supplementary Table 7. Health behavior change before and after intervention (Missing value was imputed with mean value.) 14](#_Toc209310044)

[Supplementary Table 8. Subgroup analysis of knowledge score before and after intervention 16](#_Toc209310045)

[Supplementary Table 9 Subgroup analysis of attitude score before and after intervention (Missing data were imputed with mean value) 17](#_Toc209310046)

[Supplementary Table 10. Subgroup analysis of behavior score before and after intervention (Missing data were imputed with mean value) 18](#_Toc209310047)

[Supplementary Table 11ㄡ Effect size of digital versus non-digital case management on SBP, knowledge, attitude and behavior 19](#_Toc209310048)

[Supplementary Figure 2. Subgroup analysis of BP change after intervention 20](#_Toc209310049)

[Supplementary Figure 3. Subgroup analysis comparing the effect size of digital vs. non-digital case management on knowledge, attitude, and behavior improvement 22](#_Toc209310050)

[III. Sensitivity analysis for imputing average value to missing values in attitude and health behavior score 24](#_Toc209310051)

[Supplementary Table 12. Score of attitude and behavior in pre and post-test in overall study population without imputing average value to missing values in attitude or health behavior score 24](#_Toc209310052)

[Supplementary Table 13. Subgroup analysis of attitude score before and after intervention without imputing average value to missing values in attitude score 25](#_Toc209310053)

[Supplementary Table 14. Subgroup analysis of health behavior score before and after intervention without imputing average value to missing values in health behavior score 26](#_Toc209310054)

[Supplementary Table 15. Subgroup analysis comparing the effect size of digital vs. non-digital case management on attitude without imputing average value to missing values in attitude score 27](#_Toc209310055)

[Supplementary Table 16. Subgroup analysis comparing the effect size of digital vs. non-digital case management on health behavior without imputing average value to missing values in health behavior score 28](#_Toc209310056)

[Reference 29](#_Toc209310057)

I. Supplemental methods

| Supplementary Table 1. Questions in questionnaire |
| --- |
| Knowledge |
| 1. Do you know what the normal BP values are? 2. Do you know what the 7-2-2 principle for HBPM is? 3. Do you know the time points for morning and evening measurements in the 722 principle for HBPM? |
| Attitudes |
| 1. I am willing to follow the '7-2-2 principle' for measuring BP. 2. I am willing to persuade friends and family to measure BP regularly. 3. I am willing to follow professional advice to adjust my healthy lifestyle. 4. I will actively seek professional medical advice if my BP >130/80 mmHg. 5. Reminders from friends and family will increase my willingness to measure BP regularly. 6. Reminders from professionals will increase my willingness to measure BP regularly. 7. I believe that participating in this waist circumference and BP monitoring program will contribute to better health management. |
| Health behavior change |
| 1. I measure my BP in the morning and evening. 2. I do not smoke. 3. I do not consume alcohol. 4. I pay attention to maintaining an ideal body weight. 5. I limit my intake of high-sodium foods, including pickled and canned foods. 6. I try to consume more vegetables, fruits, grains, and low-fat foods. 7. I engage in regular physical exercise. 8. I monitor my BP control to prevent complications. |

| A  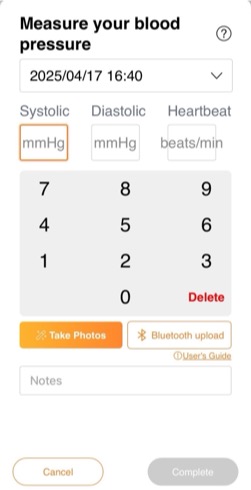 | B  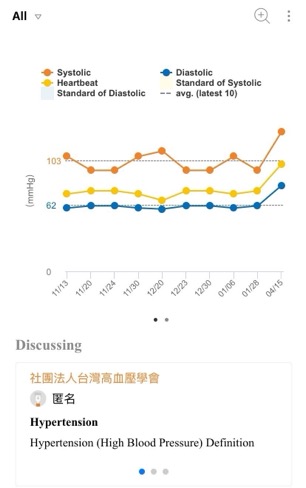 |
| --- | --- |
| C  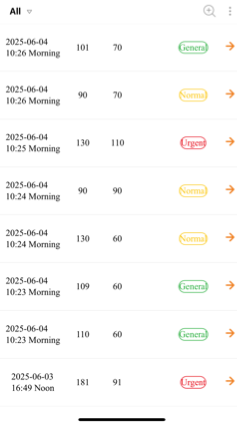 | D  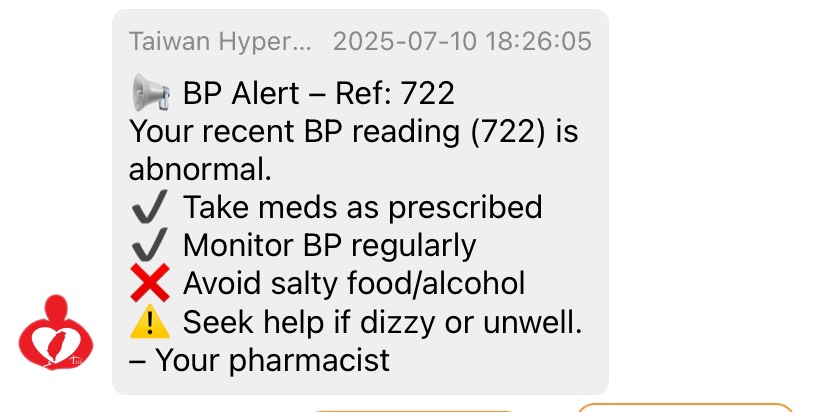 |
| Supplementary Figure 1. Schematic diagram of the HBP telemonitoring application launched on the WaCare platform  **A. Manual Entry Interface** This interface enables users to manually input blood pressure, heart rate, and measurement time. Optional features include photo upload and note entry. Its simple design promotes usability and encourages consistent data entry, crucial for accurate self-tracking in digital health contexts. The BP data also uploads automatically via Bluetooth.  **B. Trend Analysis of SBP, DBP, and heart rate**  This time-series plot illustrates trends in systolic and diastolic pressures, and heart rate, against clinical thresholds. A 10-measurement moving average aids in identifying deviations, supporting early risk detection.  **C. Blood Pressure Log Summary** This figure shows a sequential log of systolic/diastolic BP and heart rate readings. Status tags ("Normal", "General“, “Urgent", ) assist in quick health assessment. This structured log supports routine self-monitoring.  **D. Reminder, Real-time feedback and consultation to the pharmacists**  This figure shows the reminder, real-time feedback and consultation to the pharmacists after analysis of systolic/diastolic BP and heart rate readings. The content is sent to users via SMS. | |

| Supplementary Table 2. Criteria for the pharmacy to refer patients to the clinics | | | |
| --- | --- | --- | --- |
| **Home Blood Pressure Monitoring** | **Medication Use** | **Risk  Factors** | **Recommendations** |
| ≥130/80 mmHg | yes |  | May refer to clinics for drug adjustment |
| ≥130/80 mmHg &  <140/90 mmHg | no | ≥3 | Suggest referral to clinics and initiating medication |
|  |  | <3 | After over three months of follow-up and lifestyle changes, if blood pressure remains ≥130/80 mmHg, consider referral to clinics and initiating medication. |
| ≥140/90 mmHg | no |  | Suggest referral to clinics and initiating medication |
| Risk factor: Age ≥65, Male gender, Dyslipidemia, Smoking, Family history of early-onset (<50 years) cardiovascular disease, Pregnancy-induced hypertension, Preeclampsia, Adverse pregnancy outcomes | | | |

**Questionnaire design**

The present questionnaire was revised by three senior scholars from Ministry of Health and Welfare who were familiar with hypertension prevention and questionnaire design. The importance, applicability, text clarity and validity were reviewed by three experts. We then adjusted the questions according to their professional opinion.

To accommodate the questionnaire completion tendencies of individuals from diverse regions and socioeconomic strata, both online and paper questionnaires were provided for respondents to fill in. However, the filling methods of both questionnaires were the same - except for the knowledge-related questions, which were filled in by the pharmacist based on whether the answers were correct or not, other questions were self-filled.

**Classification of urbanization and digitalization of pharmacy location**

The urbanization level of the area in which a pharmacy is located was categorized as follows: high (central cities, industrial and commercial zones), medium (newly developed towns, traditional industrial towns), and low (underdeveloped towns, aging towns, and remote areas) , based on the categorization proposed in *The Typology of Townships in Taiwan:The Analysis of Sampling Stratification of the 2005-2006"Taiwan Social Change Survey”* (Pei-Chun Hou, et al, 2008).[1]

The digitalization level of the area was defined based on the classification proposed in the *Study on the Classification of Digital Development in Townships, Cities, and Districts* by the Research, Development and Evaluation Commission, Executive Yuan (Taipei, Taiwan, 2012). Digitalization was evaluated across six domains: human resource structure, socio-economic development, educational and cultural development, transportation infrastructure, living environment, and information infrastructure. Areas were classified into five levels according to these indicators, with Level 1 representing the highest level of digital development and Level 5 the lowest. In the present study, Level 1 was defined as high digitalization, Level 2 as medium digitalization, and Level 3 to 5 as low digitalization.

II. Supplemental Results

|  |
| --- |
| 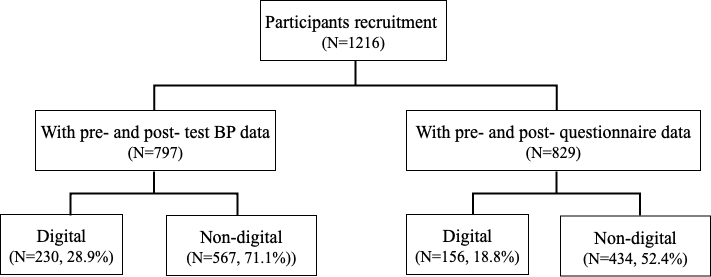 |
| Supplementary Figure 2. Flowchart of participants based on 1) who didn’t completely miss pre-test or post-test BP data or who didn’t completely miss pre-test or post-test questionnaire data 2) digital with telemonitoring or non-digital case management |

| Supplementary Table 3. Linear mixed-effects model of systolic blood pressure over time and its association with lifestyle changes and medication adherence | | | | |
| --- | --- | --- | --- | --- |
| Independent variables | β  (main effect) | P value | β  (interaction with time) | P-interaction |
| Hypertension | 17.512 | <0.001 |  | 0.329 |
| Drug adherence |  | 0.349 | -8.397 | <0.001 |
| No alcohol intake |  | 0.14 |  | 0.307 |
| Decreased salt intake |  | 0.24 |  | 0.573 |
| Increased vegetable consumption |  | 0.838 |  | 0.403 |
| Increased exercise |  | 0.078 |  | 0.608 |
| Age | 0.191 | <0.001 |  |  |
| Sex | 2.867 | <0.001 |  |  |
| Time |  | 0.345 |  |  |
| The linear mixed model used blood pressure as dependent variable. This model was adjusted to age and sex. β were calculated only when P value or P-interaction was <0.05.  *P value indicates the significance main effect of each independent variables on blood pressure.  *P-interaction tests the significance for interaction term between independent variables and time, assessing whether changes in blood pressure across time vary by levels of that independent variable. | | | | |

| Supplementary Table 4. Multiple Logistic Regression Analysis for Factors Associated with digital or non-digital case management | | |
| --- | --- | --- |
| Variable | Odds ratio (95% CI) | P value |
| Age | 1.019(1.005 to 1.034) | 0.010 |
| Sex | 0.926(0.615 to 1.384) | 0.072 |
| Educational level | 0.595 (0.468 to 0.755) | <0.001 |
| Urbanization | 1.540 (1.134 to 2.091) | 0.006 |
| Digitalization | 0.943(0.739 to 1.202) | 0.635 |
| Hypertension before intervention | 1.163 (0.772 to 1.753) | 0.470 |
| Odds ratio greater than 1 indicated choosing non-digital over digital case management. Hypertension before intervention was defined as initial home BP measured ≥ 130/80mmHg. Educational level is stratified in 3 groups (Low, medium, high. Higher level denoted higher categorial number). Urbanization and digitalization level of participating pharmacy location are stratified in 3 groups (Low, medium, high. Higher level denotes lower categorial number). | | |

| Supplementary Table 5. Proportion of correct responses for knowledge before and after intervention | | | | |
| --- | --- | --- | --- | --- |
| Questions | Proportion of correct responses | | Proportion increase  (post-test - pre-test) | *P value |
|  | Pre-test | Post-test |  |  |
| 1. Do you know what the normal BP values are? | 44.7% | 82.1% | 37.4% | <0.001 |
| 2. Do you know what the 7-2-2 protocol for HBP monitoring is? | 28.9% | 81.0% | 52.1% | <0.001 |
| 3. Do you know the time points for morning and evening measurements in the 7-2-2 protocol for HBP monitoring? | 35.4% | 82.9% | 47.5% | <0.001 |
| Scoring method: Responses evaluated by the pharmacist as 'completely correct' are considered correct, while 'partially correct' or 'unknown' responses are considered incorrect.  *McNemar test  BP: blood pressure; HBP: home blood pressure | | | | |

| Supplementary Table 6. Attitude change before and after intervention (Missing value was imputed with mean value.) | | | | | | |
| --- | --- | --- | --- | --- | --- | --- |
| Questions | Pre-test score | Post-test score | Score difference | P value | The proportion of imputed data relative to the total dataset in each question | |
|  |  |  |  |  | Pre-test | Post-test |
| 1. I am willing to follow the '7-2-2 principle' for measuring BP. | 7.95 | 8.78 | 0.83 | <0.001 | 0.8% | 2.0% |
| 2. I am willing to persuade friends and family to measure BP regularly. | 7.49 | 8.61 | 1.12 | <0.001 | 0% | 2.0% |
| 3. I am willing to follow professional advice to adjust my healthy lifestyle. | 8.23 | 8.61 | 0.66 | <0.001 | 0% | 0.5% |
| 4. I will actively seek professional medical advice if my BP >130/80 mmHg. | 8.08 | 8.95 | 0.88 | <0.001 | 0.8% | 2.0% |
| 5. Reminders from friends and family will increase my willingness to measure BP regularly. | 7.87 | 8.81 | 0.94 | <0.001 | 1.0% | 2.0% |
| 6. Reminders from professionals will increase my willingness to measure BP regularly. | 8.34 | 9.12 | 0.77 | <0.001 | 0.8% | 2.0% |
| 7. I believe that participating in this waist circumference and BP monitoring program will contribute to better health management. | 8.46 | 9.16 | 0.70 | <0.001 | 0.8% | 2.0% |
| Scoring method: 1-10 points, where 1 represents 'strongly disagree' and 10 represents 'strongly agree'.  *paired t-test  BP: blood pressure | | | | | | |

| Supplementary Table 7. Health behavior change before and after intervention (Missing value was imputed with mean value.) | | | | | | |
| --- | --- | --- | --- | --- | --- | --- |
| Questions | Pre-test score | Post-test score | Score difference | P value | The proportion of imputed data relative to the total dataset in each question | |
|  |  |  |  |  | Pre-test | Post-test |
| 1. I measure my BP in the morning and evening. | 2.58 | 3.76 | 1.19 | <0.001 | 0.8% | 1.3% |
| 2. I do not smoke. | 4.59 | 4.70 | 0.10 | 0.058 | 0.5% | 1.2% |
| 3. I do not consume alcohol. | 4.60 | 4.74 | 0.14 | 0.002 | 0.1% | 1.3% |
| 4. I pay attention to maintaining an ideal body weight. | 3.63 | 4.12 | 0.49 | <0.001 | 0.5% | 0.5% |
| 5. I limit my intake of high-sodium foods, including pickled and canned foods. | 3.80 | 4.21 | 0.41 | <0.001 | 0.1% | 0.5% |
| 6. I try to consume more vegetables, fruits, grains, and low-fat foods. | 3.97 | 4.33 | 0.36 | <0.001 | 0.1% | 1.2% |
| 7. I engage in regular physical exercise. | 3.17 | 3.64 | 0.47 | <0.001 | 0.1% | 1.3% |
| 8. I monitor my BP control to prevent complications. | 3.41 | 4.24 | 0.82 | <0.001 | 0.4% | 1.4% |
| Scoring method for questions 1 to 8: For the past 3 months, rate how frequently you have engaged in the following health behaviors each week on a scale of 1-5, where 1 = did not do it, 2 = did it 1-2 days, 3 = did it 3-4 days, 4 = did it 5-6 days, and 5 = did it all 7 days.  *paired t-test  BP: blood pressure | | | | | | |

Table S4, S5, S6 present detailed score improvement in each questionnaire items. Notably, there was a significant increase of participants recognizing 7-2-2 protocol for HBP monitoring from 28.9% before intervention to 81.0% afterward. Additionally, participants more frequently measured their BP in the morning and evening, with score increased from 2.58 to 3.76. Participants more frequently monitored their BP to prevent cardiovascular complications, with score increased from 3.41 to 4.24. The proportion of imputed data relative to the total dataset in each question is quite low, less than or equal to 2%.

| Supplementary Table 8. Subgroup analysis of knowledge score before and after intervention | | | |
| --- | --- | --- | --- |
|  | Pre-test (N=829) | Post-test (N=829) | Difference |
| Age |  |  |  |
| <40 | 1.5 | 2.5 | **1.0** |
| 40-64 | 1.2 | 2.5 | **1.3** |
| ≥65 | 0.9 | 2.4 | **1.5** |
| P value |  | 0.149 | *0.031 |
| Educational level |  |  |  |
| Low | 0.8 | 2.4 | **1.6** |
| Medium | 1.2 | 2.5 | **1.3** |
| High | 1.4 | 2.5 | **1.1** |
| P value |  | 0.248 | 0.067 |
| Urbanization |  |  |  |
| Low | 0.9 | 2.5 | **1.6** |
| Medium | 1.0 | 2.5 | **1.5** |
| High | 1.3 | 2.4 | **1.2** |
| P value |  | 0.727 | *0.007 |
| Digitalization |  |  |  |
| Low | 1.09 | 2.43 | **1.33** |
| Medium | 1.04 | 2.50 | **1.46** |
| High | 1.15 | 2.49 | **1.35** |
| P value |  | 0.718 | 0.634 |
| The P value represents the statistical significance of the difference in post-test scores or the change in scores from pre-test to post-test within each category. The words in bold indicate a significant difference between pre- and post-test scores. | | | |

| Supplementary Table 9 Subgroup analysis of attitude score before and after intervention (Missing data were imputed with mean value) | | | |
| --- | --- | --- | --- |
|  | Pre-test (N=829) | Post-test (N=829) | Difference |
| Age |  |  |  |
| <40 | 57.7 | 61.5 | **3.8** |
| 40-64 | 57.5 | 62.2 | **4.8** |
| ≥65 | 54.7 | 62.7 | **8.0** |
| P value |  | 0.638 | *0.018 |
| Educational level |  |  |  |
| Low | 52.7 | 61.7 | **9.0** |
| Medium | 57.3 | 62.7 | **5.4** |
| High | 59.5 | 60.6 | 1.1 |
| P value |  | 0.290 | *0.003 |
| Urbanization |  |  |  |
| Low | 53.0 | 62.2 | **9.2** |
| Medium | 54.9 | 61.8 | **6.8** |
| High | 59.6 | 63.0 | **3.4** |
| P value |  | 0.340 | * 0.001 |
| Digitalization |  |  |  |
| Low | 56.4 | 62.0 | **5.6** |
| Medium | 56.1 | 62.3 | **6.2** |
| High | 56.7 | 62.6 | **5.9** |
| P value |  | 0.802 | 0.926 |
| The P value represents the statistical significance of the difference in post-test scores or the change in scores from pre-test to post-test within each category. The words in bold indicate a significant difference between pre- and post-test scores. | | | |

| Supplementary Table 10. Subgroup analysis of behavior score before and after intervention (Missing data were imputed with mean value) | | | |
| --- | --- | --- | --- |
|  | Pre-test (N=829) | Post-test (N=829) | Difference |
| Age |  |  |  |
| <40 | 28.7 | 33.4 | **4.7** |
| 40-64 | 29.6 | 33.6 | **4.0** |
| ≥65 | 30.3 | 34.0 | **3.7** |
| P value |  | 0.430 | 0.666 |
| Educational level |  |  |  |
| Low | 29.1 | 33.6 | **4.5** |
| Medium | 29.9 | 33.8 | **3.9** |
| High | 29.6 | 33.3 | **3.6** |
| P value |  | 0.735 | 0.684 |
| Urbanization |  |  |  |
| Low | 30.9 | 34.0 | **3.1** |
| Medium | 28.4 | 33.4 | **5.0** |
| High | 30.7 | 34.0 | **3.3** |
| P value |  | 0.411 | 0.009 |
| Digitalization |  |  |  |
| Low | 29.5 | 33.3 | **3.8** |
| Medium | 29.5 | 33.8 | **4.3** |
| High | 30.2 | 34.0 | **3.8** |
| P value |  | 0.329 | 0.715 |
| The P value represents the statistical significance of the difference in post-test scores or the change in scores from pre-test to post-test within each category. The words in bold indicate a significant difference between pre- and post-test scores. | | | |

| Supplementary Table 11ㄡ Effect size of digital versus non-digital case management on SBP, knowledge, attitude and behavior | | | |
| --- | --- | --- | --- |
|  | Effect size | 95% CI | P value |
| SBP | -1.5mmHg | (-3.9~0.9) | 0.227 |
| DBP | -1.1mmHg | (-2.2~0.2) | 0.045 |
| Knowledge | -0.1 | (-0.4~0.2) | 0.622 |
| Attitude(imputed) | -2.6 | (-5.5~0.6) | 0.109 |
| Attitude(non-imputed) | -2.4 | (-5.5~0.6) | 0.120 |
| Behaviors(imputed) | -1.7 | (-3.3~ -0.0) | 0.047 |
| Behaviors(non-imputed) | -1.7 | (-3.3~-0.1) | 0.040 |
| Changes in SBP/DBP were calculated as post-test minus pre-test SBP/DBP after adjustment of baseline BP status. Thus, a negative value indicates effective BP reduction and improvement. The effect size is defined as the increase in score achieved through digital case management minus the increase in score achieved through non-digital case management. “Imputed” refers to analyses in which missing values were replaced with the average score prior to analysis, whereas “non-imputed” refers to analyses conducted without imputing missing values. | | | |

| 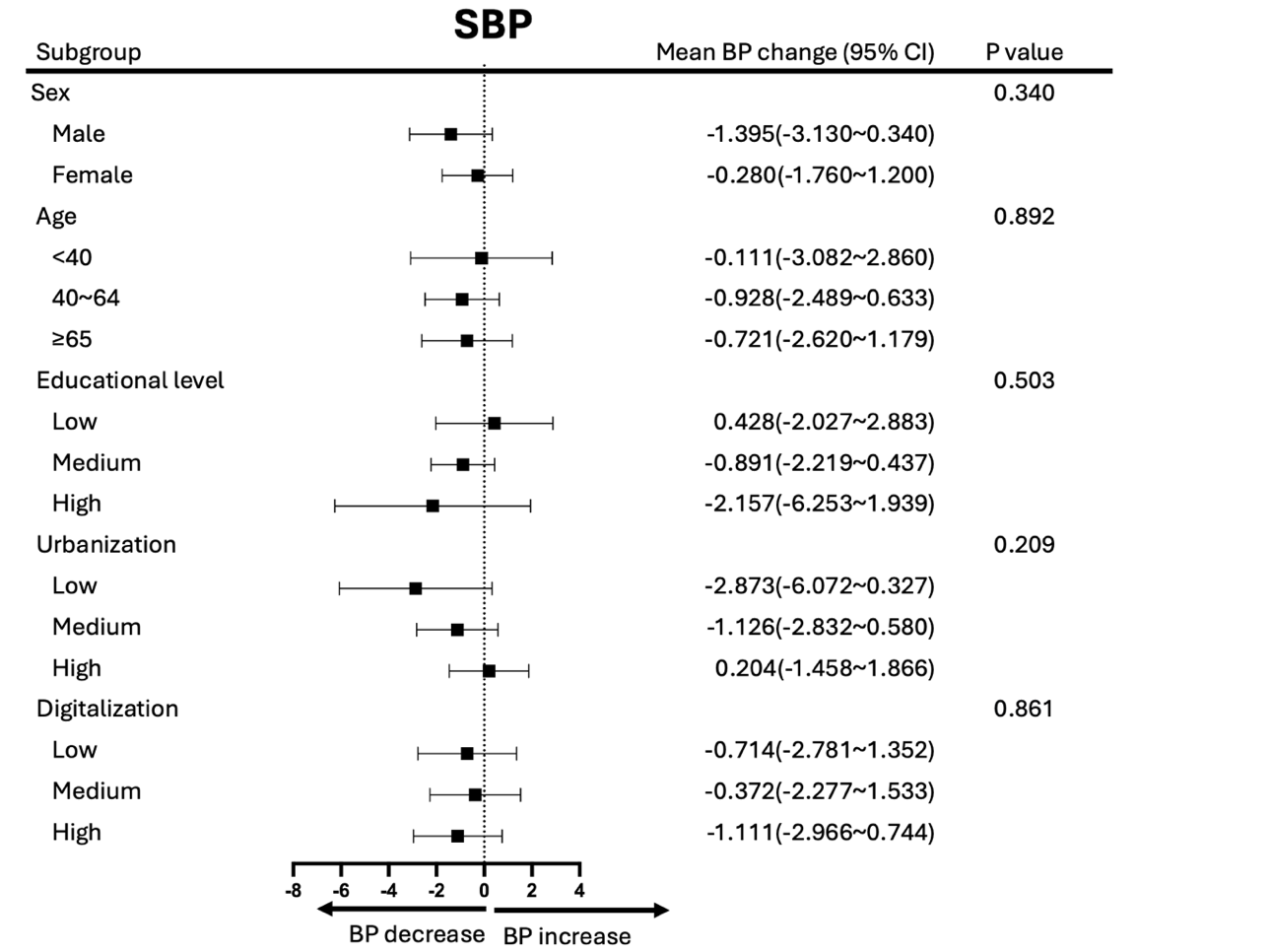  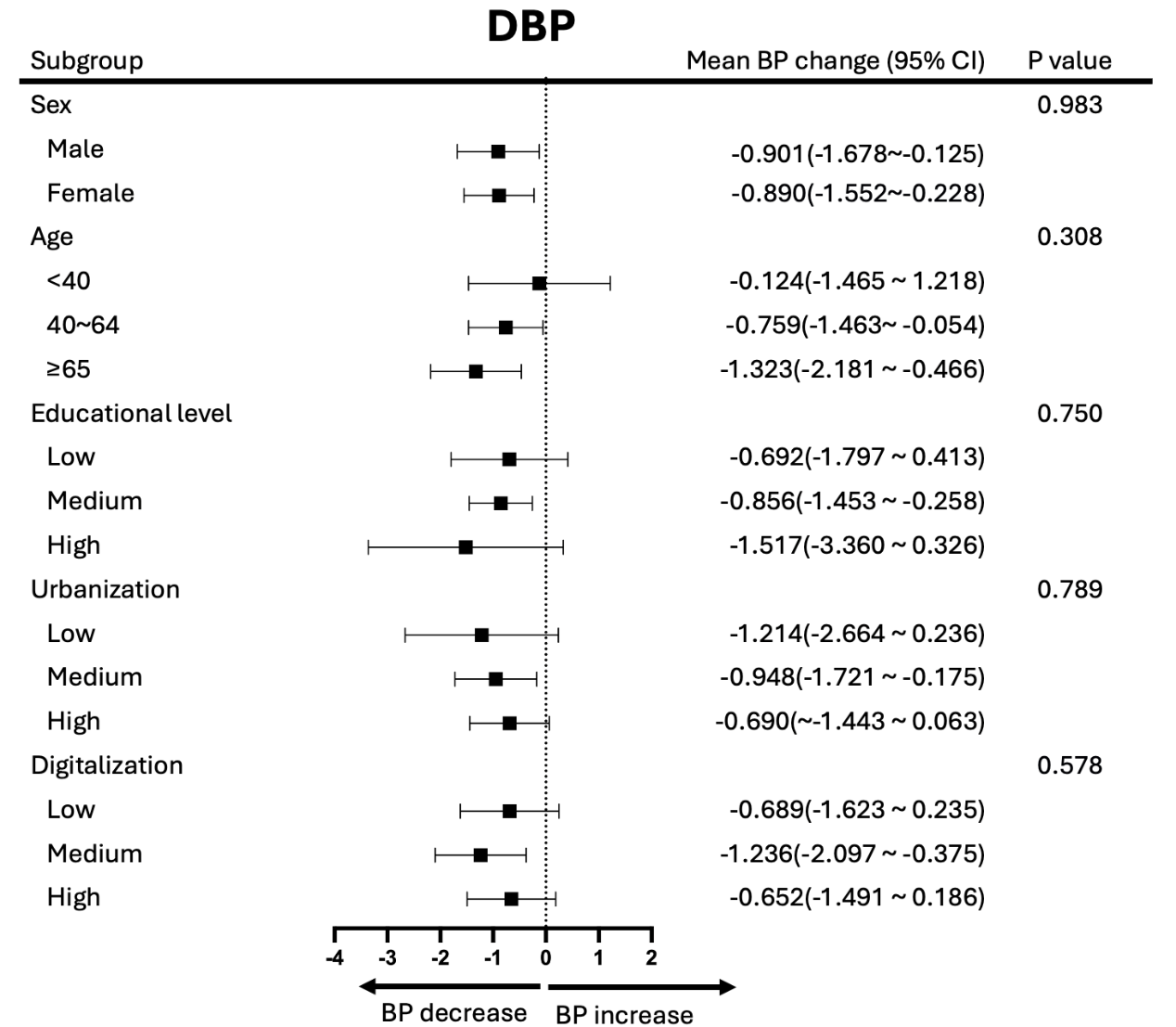 |
| --- |
| Supplementary Figure 2. Subgroup analysis of BP change after intervention  BP change is defined as the last Home BP in the whole study period minus first Home BP measured before intervention. Age is measured in years. Baseline BP status was adjusted before subgroup analysis. The black dots represent the mean effect size for each subgroup, while the gray horizontal lines indicate the 95% confidence interval. P value indicates the significance of within-subgroup difference in BP change. |

| 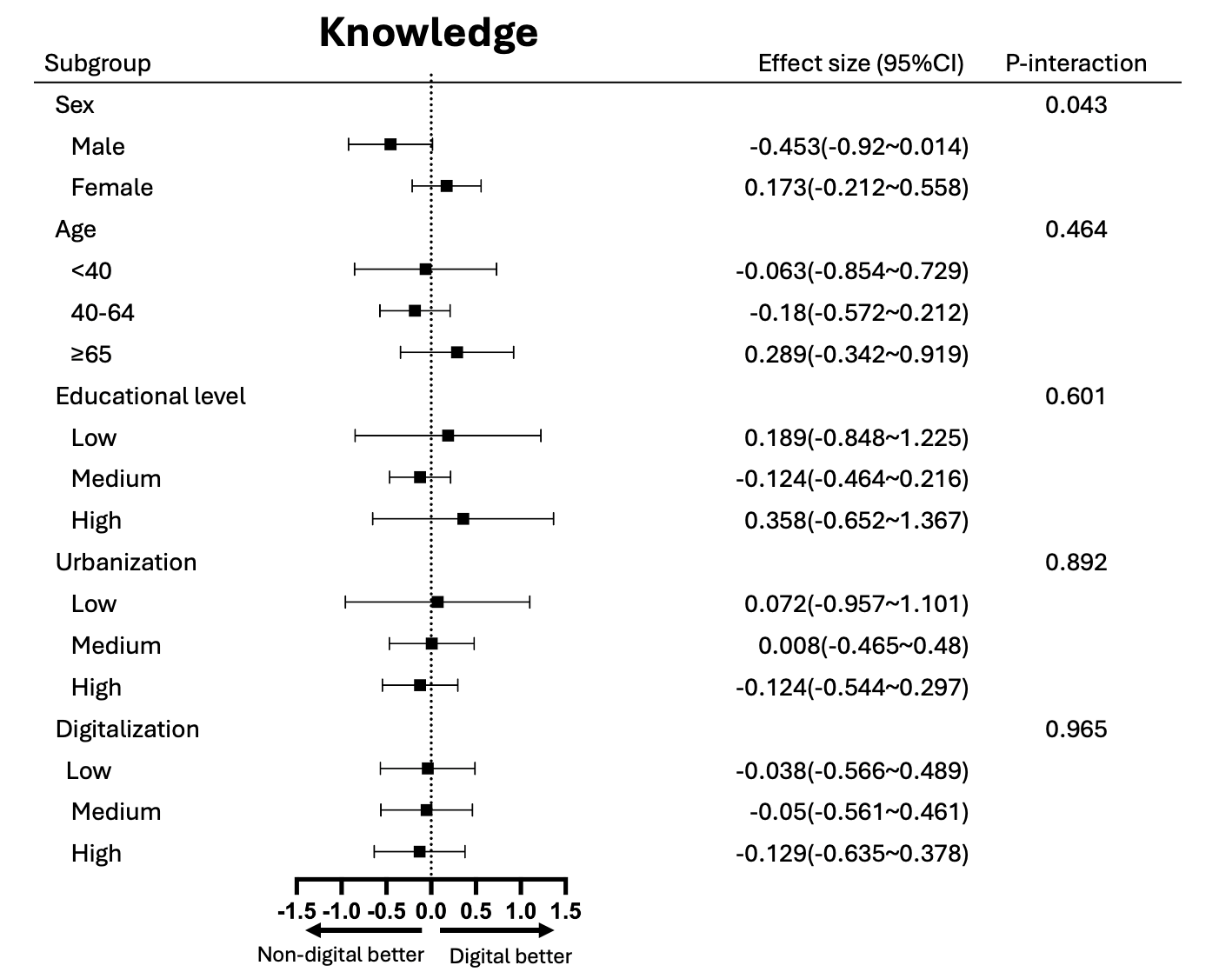  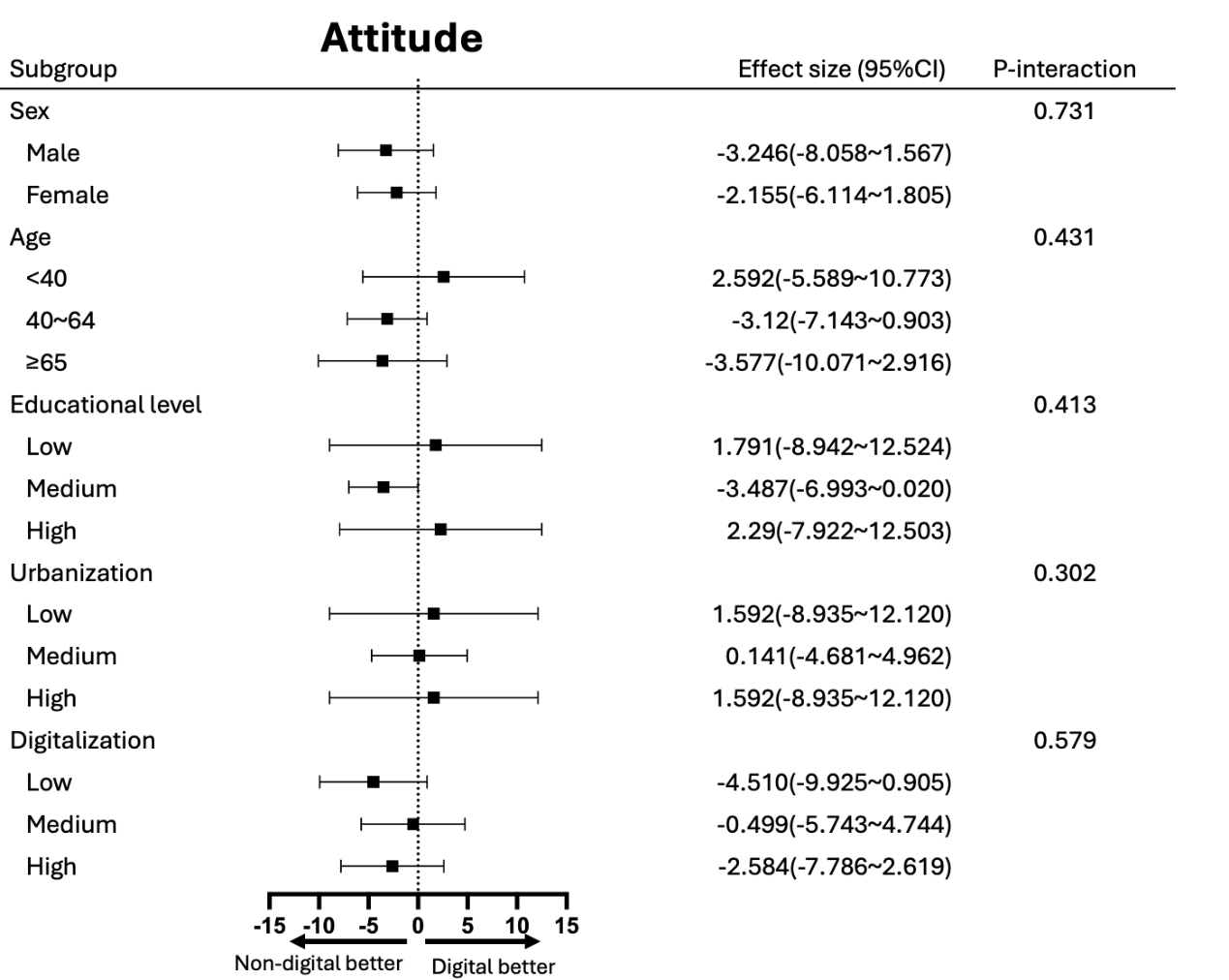  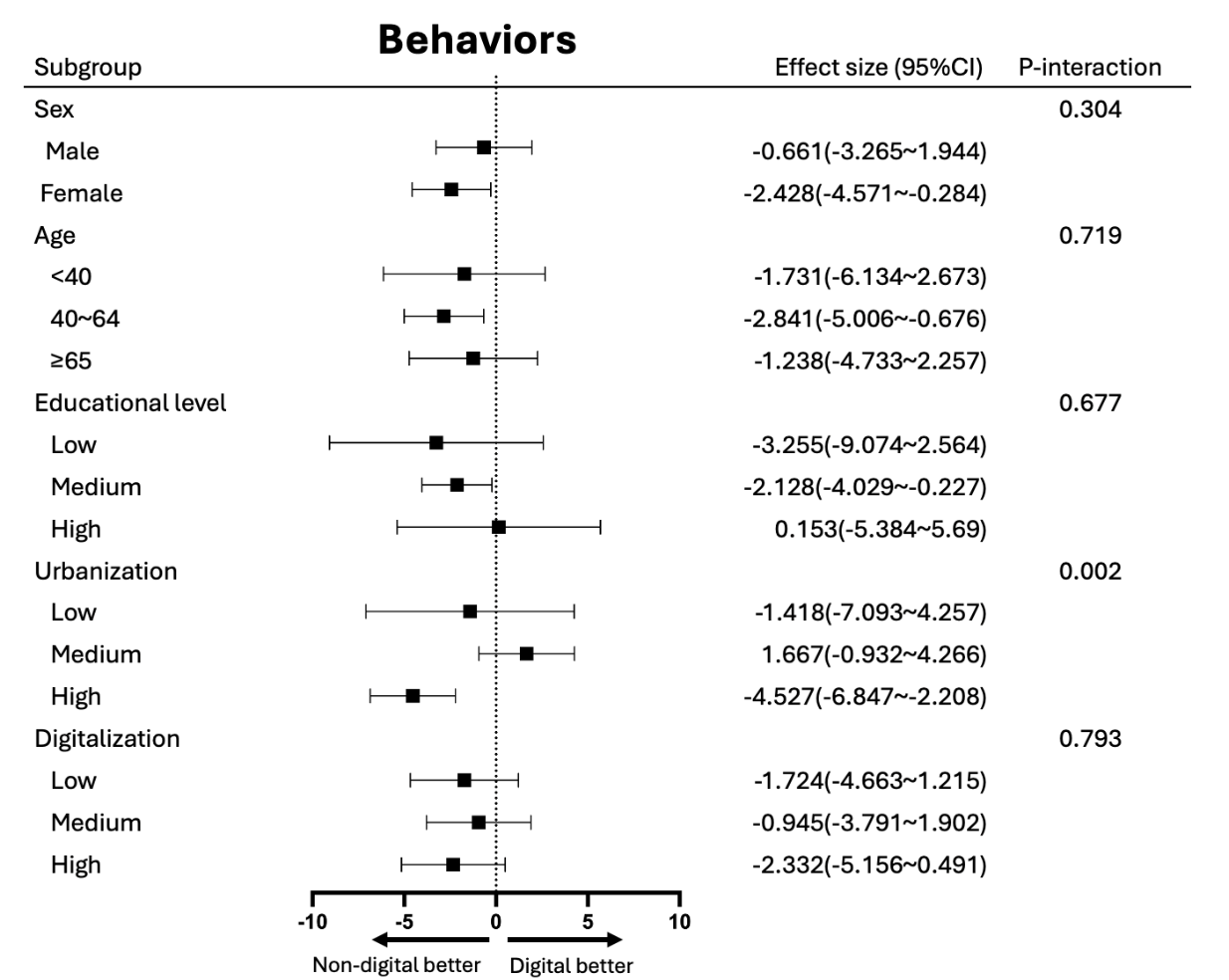 |
| --- |
| Supplementary Figure 3. Subgroup analysis comparing the effect size of digital vs. non-digital case management on knowledge, attitude, and behavior improvement  This figure illustrates the difference in the effects of digital versus non-digital case management. The effect size is defined as the increase in score achieved through digital case management minus that achieved through non-digital case management. Age is measured in years. The black dots represent the mean effect size for each subgroup, while the horizontal lines indicate the 95% confidence interval (CI). |

III. Sensitivity analysis for imputing average value to missing values in attitude and health behavior score

| Supplementary Table 12. Score of attitude and behavior in pre and post-test in overall study population without imputing average value to missing values in attitude or health behavior score | | | | |
| --- | --- | --- | --- | --- |
|  | Pre-test score | Post-test score | Difference | P value |
| Attitude | 56.9 | 62.5 | 5.6 | <0.001 |
| Behaviors | 29.7 | 33.7 | 4.0 | <0.001 |

| Supplementary Table 13. Subgroup analysis of attitude score before and after intervention without imputing average value to missing values in attitude score | | | |
| --- | --- | --- | --- |
|  | Pre-test | Post-test | Difference |
| Age |  |  |  |
| <40 | 57.8 | 61.6 | **3.8** |
| 40-64 | 57.7 | 62.5 | **4.8** |
| ≥65 | 54.8 | 62.8 | **8.0** |
| P value |  | 0.646 | *0.018 |
| Educational level |  |  |  |
| Low | 52.8 | 62.0 | **9.2** |
| Medium | 57.5 | 62.9 | **5.4** |
| High | 59.1 | 60.7 | 1.6 |
| P value |  | 0.303 | *0.004 |
| Urbanization |  |  |  |
| Low | 53.1 | 62.4 | **9.3** |
| Medium | 55.2 | 62.0 | **6.8** |
| High | 59.7 | 63.2 | **3.5** |
| P value |  | 0.328 | *0.002 |
| Digitalization |  |  |  |
| Low | 56.6 | 62.0 | **5.4** |
| Medium | 56.3 | 62.5 | **6.2** |
| High | 56.8 | 62.9 | **6.1** |
| P value |  | 0.718 | 0.842 |
| The P value represents the statistical significance of the difference in post-test scores or the change in scores from pre-test to post-test within each category. Age is measured in years. The words in bold indicate a significant difference between pre- and post-test scores. | | | |

| Supplementary Table 14. Subgroup analysis of health behavior score before and after intervention without imputing average value to missing values in health behavior score | | | |
| --- | --- | --- | --- |
|  | Pre-test | Post-test | Difference |
| Age |  |  |  |
| <40 | 28.5 | 33.4 | **4.9** |
| 40-64 | 29.6 | 33.6 | **4.0** |
| ≥65 | 30.3 | 34.1 | **3.8** |
| P value |  | 0.477 | 0.549 |
| Educational level |  |  |  |
| Low | 29.1 | 33.6 | **4.5** |
| Medium | 29.9 | 33.8 | **3.9** |
| High | 29.4 | 33.4 | **4.0** |
| P value |  | 0.856 | 0.660 |
| Urbanization |  |  |  |
| Low | 30.8 | 33.9 | **3.1** |
| Medium | 28.2 | 33.4 | **5.2** |
| High | 30.9 | 34.1 | **3.2** |
| P value |  | 0.409 | *0.008 |
| Digitalization |  |  |  |
| Low | 29.4 | 33.2 | **3.8** |
| Medium | 29.5 | 33.9 | **4.4** |
| High | 30.2 | 34.1 | **3.9** |
| P value |  | 0.248 | 0.771 |
| The P value represents the statistical significance of the difference in post-test scores or the change in scores from pre-test to post-test within each category. Age is measured in years. The words in bold indicate a significant difference between pre- and post-test scores. | | | |

| Supplementary Table 15. Subgroup analysis comparing the effect size of digital vs. non-digital case management on attitude without imputing average value to missing values in attitude score | | | | |
| --- | --- | --- | --- | --- |
| Subgroup | Mean effect size | Lower | Upper | P for interaction |
| Sex |  |  |  | 0.742 |
| Male | 3.171 | -1.684 | 8.026 |  |
| Female | 2.117 | -1.884 | 6.119 |  |
| Age |  |  |  | 0.421 |
| <40 | -2.794 | -11.097 | 5.510 |  |
| 40~64 | 3.112 | -0.956 | 7.180 |  |
| ≥65 | 3.497 | -3.039 | 10.032 |  |
| Educational level |  |  |  | 0.429 |
| Low | -1.771 | -12.570 | 9.028 |  |
| Medium | 3.409 | -0.134 | 6.952 |  |
| High | -2.290 | -12.545 | 7.964 |  |
| Urbanization |  |  |  | 0.311 |
| Low | -1.481 | -12.088 | 9.126 |  |
| Medium | -0.239 | -5.104 | 4.627 |  |
| High | 4.331 | -0.013 | 8.676 |  |
| Digitalization |  |  |  | 0.636 |
| Low | 4.151 | -1.311 | 9.613 |  |
| Medium | 0.494 | -4.812 | 5.800 |  |
| High | 2.703 | -2.543 | 7.948 |  |
| The effect size is defined as the increase in score achieved through digital case management minus the increase in score achieved through non-digital case management. Age is measured in years.  Lower indicates lower limit of 95% confidence interval. Upper indicates upper limit of 95% confidence interval. | | | | |

| Supplementary Table 16. Subgroup analysis comparing the effect size of digital vs. non-digital case management on health behavior without imputing average value to missing values in health behavior score | | | | |
| --- | --- | --- | --- | --- |
| Subgroup | Mean effect size | Lower | Upper | P for interaction |
| Sex |  |  |  | 0.364 |
| Male | 0.848 | -1.767 | 3.463 |  |
| Female | 2.416 | 0.260 | 4.573 |  |
| Age |  |  |  | 0.768 |
| <40 | 2.303 | -2.149 | 6.756 |  |
| 40~64 | 2.832 | 0.653 | 5.011 |  |
| ≥65 | 1.308 | -2.193 | 4.810 |  |
| Educational level |  |  |  | 0.664 |
| Low | 3.379 | -2.454 | 9.212 |  |
| Medium | 2.181 | 0.269 | 4.094 |  |
| High | -0.192 | -5.876 | 5.492 |  |
| Urbanization |  |  |  | 0.003 |
| Low | 1.274 | -4.415 | 6.963 |  |
| Medium | -1.510 | -4.123 | 1.103 |  |
| High | 4.588 | 2.256 | 6.920 |  |
| Digitalization |  |  |  | 0.763 |
| Low | 1.789 | -1.159 | 4.738 |  |
| Medium | 0.955 | -1.908 | 3.819 |  |
| High | 2.463 | -0.378 | 5.340 |  |
| The effect size is defined as the increase in score achieved through digital case management minus the increase in score achieved through non-digital case management. Age is measured in years. Lower indicates lower limit of 95% confidence interval. Upper indicates upper limit of 95% confidence interval. | | | | |

The score differences, subgroup analyses, mean effect sizes, and ANCOVA results from the dataset without imputed values were consistent with those from the dataset with imputed values, indicating robust findings in the sensitivity analysis.

Reference

1. Pei-Chun Hou S-HT, Pei-shan Liao, Yung-Tai Hung, Ying-Hwa Chang. The Typology of Townships in Taiwan:The Analysis of Sampling Stratification of the 2005-2006"Taiwan Social Change Survey". *Survey Research — Method and Application.* 2008.)(23):7-32.
